# Expansion of Splice-Switching Therapy with Antisense Oligonucleotides

**DOI:** 10.3390/ijms26052270

**Published:** 2025-03-04

**Authors:** Yasuhiro Takeshima

**Affiliations:** Department of Pediatrics, Hyogo Medical University, 1-1 Mukogawacho, Nishinomiya 663-8501, Japan; ytake@hyo-med.ac.jp; Tel.: +81-798-45-6350

**Keywords:** splice-switching therapy, antisense oligonucleotide, monogenic disease, non-monogenic disease

## Abstract

Since 2016, splice-switching therapy, in which splicing is controlled by antisense oligonucleotides, has been applied in clinical practice for spinal muscular atrophy and Duchenne muscular dystrophy. In the former disease, this therapy induces exon inclusion, while, in the latter, it induces exon skipping, leading expression of functional proteins. Basic and clinical studies of splice-switching therapy for many monogenic diseases have now been conducted. The molecular mechanisms of splice-switching therapy include not only the induction of exon inclusion and skipping, but also the induction of pseudoexon skipping and suppression of splicing sites generated by mutations. In addition, therapies that alter protein function by regulating splicing are being investigated not only for monogenic diseases but also for non-monogenic ones such as cancer and immune-related disorders. It is expected that many of these basic studies will be translated into clinical applications. This review describes the current status of basic research and clinical applications of splice-switching therapy to promote the development of treatments for noncurable diseases.

## 1. Introduction

Genomic information is transcribed into mRNA precursors, which are then subjected to post-transcriptional modifications such as splicing and translated into proteins [1]. Previously, antisense oligonucleotides were thought to be useful tools for regulating mRNA expression by RNaseH and other means. However, in 1993, it was reported that splicing abnormalities caused by point mutations in the beta-globin gene could be repaired by antisense oligonucleotide (AS-oligo) [2]. In 1995, my colleagues and I proposed a splice-switching therapy for Duchenne muscular dystrophy (DMD) by inducing exon skipping of dystrophin genes with an AS-oligo [3]. Nowadays, this therapy is clinically applied in some DMD cases [4]. Meanwhile, the range of indications for splice-switching therapy with AS-oligos is rapidly expanding and it has already been clinically applied in spinal muscular atrophy [5]. Furthermore, its application is not limited to monogenic diseases, but is also being investigated as a basic treatment for malignant and immune-related diseases [4]. In this paper, we discuss the expansion of splice-switching therapy.

## 2. From the Discovery of Splicing to the Elucidation of Molecular Mechanisms

In the genome, protein-coding regions are broken up by non-coding regions, and the step of converting the fragmented genetic information into a contiguous sequence of information is known as splicing. After genetic information is transcribed from genomic DNA to mRNA precursors, it undergoes post-transcriptional modifications to become mature mRNA, which is then translated to produce proteins. One of the major steps in post-transcriptional modification is RNA splicing. This splicing mechanism was first described by two groups in 1977 [6,7]. The selectivity of splicing is an important mechanism for the production of diverse proteins from a single gene.

As the splicing mechanism was clarified, highly conserved sequences involved in splice site determination, such as GU sequences at the 5′ end of introns, AG sequences at the 3′ end, branch points, and polypyrimidine tracts, have been identified. These sites are then recognized by the snRNP complexes and others to determine the splicing site [8]. In the 1990s, it became clear that there are *cis*-sequences within exons or introns that promote or inhibit splicing, which were termed exonic and intronic splicing enhancers (ESE and ISE) and exonic and intronic splicing silencers (ESS and ISS) [8,9,10]. Serine–arginine (SR) proteins, heterogeneous nuclear ribonucleoproteins (hnRNPs) and unknown factors bind to these sequences and regulate splicing.

Today, splicing defects caused by variants in splicing consensus sequences at exon–intron junctions or in ESEs are known to be the cause of many genetic diseases [11].

## 3. Identification of Exonic Splicing Enhancer in Dystrophin Gene and Regulation of Splicing by Antisense Oligonucleotide

In the course of genetic analysis of DMD cases, we encountered the following abnormal splicing cases. In a DMD case in which a 52-nucleotide deletion in exon 19 of the dystrophin gene was identified, the splicing consensus sequences of intron 18, exon 19, and intron 19 at the exon–intron boundary were not mutated, but exon 19 was skipped in the mRNA. We named this genetic variant “dystrophin Kobe” [12]. Although the molecular biological mechanism was unclear at that time, the importance of ESE in splicing control had just been recognized [9]. We examined the possibility that ESE was present within the 52 bases that were deleted in dystrophin Kobe and that the deletion of ESE caused the skipping of exon 19. Analysis by an in vitro splicing system using mini-genes showed that ESE was present in the deleted region of exon 19, and that the splicing was controlled by inhibiting the ESE using AS-oligo [3]. The results showed that splicing can be controlled by inhibiting the ESE using AS-oligo.

Approximately 60% of gene variants causative of DMD involve deletion of one or a few exons of the dystrophin gene. In DMD, such deletions usually result in a frame shift, causing disruption of the amino acid reading frame. Meanwhile, in the milder form, Becker muscular dystrophy (BMD), there is an in-frame deletion in which the amino acid reading frame is maintained [13]. Exon 20 deletion is an out-of-frame deletion of 242 bases, while exons 19 and 20 deletion are in-frame deletions of 330 bases (88 + 242). In other words, in DMD cases with exon 20 deletion, induction of exon 19 skipping by AS-oligo is expected to reduce the severity of symptoms. We have shown that AS-oligo induces exon 19 skipping and dystrophin protein production in exon 20-deleted DMD myocytes in a culture system [14]. Furthermore, we found that intravenous administration of AS-oligo induced dystrophin production in muscle tissue of a DMD patient [15].

Eteplirsen, an AS-oligo that induces exon 51 skipping, was approved under the accelerated approval pathway in the United States in 2016, and golodirsen, which induces exon 53 skipping, was in 2019 [16]. Moreover, in 2020, viltolarsen, which induces exon 53 skipping, was approved in the United States and Japan under the accelerated or conditional accelerated approval system, and, in 2021, casimersen, which induces exon 45 skipping, was expedited for approval in the United States [16]. Meanwhile, treatment to inhibit myostatin expression by inhibiting the function of SES in exon 1 of the myostatin gene using AS-oligo is also under investigation for DMD [17].

## 4. Expanded Treatment for Monogenic Diseases

### 4.1. Therapeutic Strategy with Splice-Switching

The treatment for DMD involves inducing the skipping of out-of-frame exons using AS-oligo to correct the amino acid reading frame. Other strategies include the induction of skipping of in-frame exons with nonsense mutations to produce mRNA that skips the nonsense mutation and retains the amino acid reading frame, induction of skipping of pseudoexons activated by intron mutations to promote native mRNA production, and induction of exon skipping to alter the protein function. Conversely, some therapeutic strategies induce exon insertion by inhibiting ESS or ISS function using AS-oligo. In addition, therapeutic strategies to induce splice-switching of genes other than the responsible gene in monogenic diseases are also being investigated [4]. Treatment strategies with splice-switching are summarized in Table 1.

### 4.2. Administration to Patients

It has become clear that splicing can be regulated by AS-oligos against intra-exon and intra-intron sequences, such as exon–intron boundary sequences and ESEs involved in splicing regulation. Nowadays, this mechanism is applied to many monogenic diseases for splice-switching therapeutic applications (Table 2). Besides DMD, as shown below, in spinal muscular atrophy (SMA), neuronal ceroid lipofuscinosis 7, ataxia-telangiectasia and Leber congenital amaurosis, AS-oligo treatment has been performed on patients in clinical practice (Table 2).

Nusinersen is currently the most widely used drug in clinical application. In SMA, the responsible gene, the *survival motor neuron* (*SMN*)*1* gene, is deleted and only the backup gene, *SMN2*, is functional. The *SMN2* gene expresses only a small amount of functional protein, owing to exon 7 skipping in approximately 90% of the mRNA. However, the inclusion of exon 7 by nusinersen, an AS-oligo for ISS in intron 7, can increase the production of functional proteins [5]. It was approved in the United States in 2016 and has now been administered to more than 10,000 patients worldwide.

Meanwhile, in monogenic diseases, mutations often differ from case to case, and some diseases require the design of AS-oligos that regulate splicing on an individual case basis. In one case of neuronal ceroid lipofuscinosis 7 (a form of Batten’s disease), an SVA (SINE-VNTR-Alu) retrotransposon insertion variant within intron 6 of the *MFSD8* (also known as *CLN7*) gene activates a latent splicing acceptor site, resulting in the insertion of intron sequence into mRNA. Milasen, which inhibits activation of new splice acceptor site, was developed for this case, administered to the patient under FDA approval, and found to be effective [18]. The “N-of-1” study, the development of a treatment for a single patient, resulted in administration to the patient just 1 year after development and is being looked at as a possible avenue for future clinical development.

AS-oligo was also administered to patients with ataxia-telangiectasia as a form of personalized medicine [19]. An AS-oligo named atipeksen, which inhibits a newly created splicing donor site resulting from a point mutation within exon 53 of the *ATM* gene, was developed and administered to an affected 2-year-old child after preclinical testing. Three-year tolerability was reported [19].

The efficacy of intravitreal administration of sepofarsen, an AS-oligo that induces pseudoexon skipping, has also been reported for Leber congenital amaurosis type 10, in which a point mutation within intron 26 of the *CEP290* gene creates a splice donor site and a pseudoexon is inserted [20].

Today, AS-oligo administration is being used in real-world clinical practice in several diseases. It is also anticipated that AS-oligos currently in the preclinical phase as described below, will be developed for clinical use in the future.

**Table 2:** Splice-switching therapy with antisense oligonucleotides for monogenic diseases (except Duchenne muscular dystrophy and spinal muscular atrophy).

Disease	Gene	Exon	Splice Switching	Phase	Author, Year
Neuromuscular disease					
LGMDR2	*DYSF*	32	ES	in vitro	Barthélémy et al., 2015 [21]
	*DYSF*	int 50	PE	in vitro	Dominov et al., 2019 [22]
LGMDR5	*SGCG*	4–7	ES	in vitro	Wyatt et al., 2018 [23]
laminopathy	*LMNA*	5	ES	in vitro	Scharner et al., 2015 [24]
Ullrich CMD	*COL6A1*	int 11	PE	in vitro	Aguti et al., 2020 [25]
FCMD	*FKTN*	int 5	PE	in vitro	Enkhjargal et al., 2023 [26]
myotonic dystrophy	*Clcn1* *	7a	ES	mouse	Hu et al., 2023 [27]
	*DMPK*	15	ES	in vitro	Stepniak-Konieczna et al., 2020 [28]
neuronal ceroid lipofuscinosis	*CLN7*	int 6	NSI	human	Kim et al., 2019 [18]
Huntington’s disease	*HTT*	12	ES	mouse	Kim et al., 2022 [29]
Dravet’s disease	*SCN1A*	20N	TANGO	mouse	Yuan et al., 2024 [30]
Parkinson’s disease	*PRKN*	4	ES	in vitro	Li et al., 2020 [31]
	*LRRK2*	41	ES	mouse	Korecka et al., 2020 [32]
Joubert syndrome	*CEP290*	41	ES	in vitro	Molinari et al., 2019 [33]
fragile X	*FMR1*	int 1	PE	in vitro	Shah et al., 2023 [34]
CADASIL	*NOTCH3*	9	ES	in vitro	Gravesteijn et al., 2020 [35]
	*NOTCH3*	2–3, 4–5, 6	ES	in vitro	Rutten et al., 2016 [36]
spinocerebellar ataxia type 3	*ATXN3*	10	ES	in vitro	McIntosh et al., 2019 [37]
BPAN	*WDR45*	int 5	PE	in vitro	Yamada et al., 2024 [38]
NF2-related schwannomatosis	*NF2*	11	ES	in vitro	Catasús et al., 2022 [39]
Timothy syndrome	*CACNA1C*	8A	ES	rat	Chen et al., 2024 [40]
Inborn error of metabolism					
glycogen storage disease 1a	*G6PC*	5	NSI	mouse	Ito et al., 2023 [41]
Pompe disease	*Gys1* *	6	ES	mouse	Clayton et al., 2014 [42]
	*GAA*	2	EI	in vitro	van der Wal et al., 2017 [43]
phenylketonuria	*PAH*	11	EI	in vitro	Martínez-Pizarro et al., 2024 [44]
propionic acidemia	*PCCA*	int 14	TANGO	in vitro	Spangsberg Petersen et al., 2023 [45]
methylmalonic aciduria	*MUT*	int 11	PE	in vitro	Pérez et al., 2009 [46]
mucolipidosis type 2	*GNPTAB*	19	ES	in vitro	Matos et al., 2020 [47]
Fabry disease	*GLA*	int 4	PE	in vitro	Palhais et al., 2016 [48]
Niemann–Pick type C	*NPC1*	int 9	PE	in vitro	Rodríguez-Pascau et al., 2009 [49]
familial hypercholesterolemia	*APOB* *	27	ES	mouse	Disterer et al., 2013 [50]
Kidney disease					
X-linked Alport syndrome	*COL4A5*	21	ES	mouse	Yamamura et al., 2020 [51]
autosomal recessive polycystic kidney disease	*PKHD1*	int 21	NSI	in vitro	Li et al., 2023 [52]
Eye disease					
Leber congenital amaurosis	*CEP290*	int 26	PE	human	Cideciyan et al., 2021 [53]
					Russell et al., 2022 [20]
Stargardt disease type 1	*ABCA4*	int 38	EI	in vitro	Kaltak et al., 2023 [54]
	*ABCA4*	int 6, 7, 13, and 30 int 28	PE EI	in vitro	Sangermano et al., 2019 [55]
retinitis pigmentosa 11	*PRPF31*	12	ES	in vitro	Grainok et al., 2024 [56]
Usher syndrome type 3	*CLRN1*	int 0b	PE	mouse	Panagiotopoulos et al., 2020 [57]
Skin disease					
xeroderma pigmentosum	*ERCC4/XPF*	int 1	NSI	in vitro	Senju et al., 2023 [58]
recessive dystrophic epidermolysis bullosa	*COL7A1*	int 51, 104	NSI, PS	in vitro	Pironon et al., 2024 [59]
	*COL7A1*	3	NSI	in vitro	Hainzl et al., 2024 [60]
	*COL7A1*	73	ES	in vitro	Bornert et al., 2021 [61]
Others					
ataxia–telangiectasia	*ATM*	53	NSI	human	Kim et al., 2023 [19]
progressive familial intrahepatic cholestasis type 2	*ABCB11*	int 2	NSI	in vitro	Zheng et al., 2022 [62]
Hutchinson–Gilford progeria	*LMNA*	11	NSI	in vitro	Abdelrahman et al., 2023 [63]
cystic fibrosis	*CFTR*	23	ES	in vitro	Kim et al., 2022 [64]
erythropoietic protoporphyria	*FECH*	int 3	NSI	mouse	Halloy et al., 2020 [65]

Genes responsible for myotonic dystrophy, Pompe disease, and familial hypercholesterolemia are *DMPK*, *GAA*, and *LDLR* (*), respectively. int: intron; ES: skipping of authentic exon; PS: skipping of pseudoexon; EI: inclusion of authentic exon; NSI: new splice site inhibition; TANGO: Targeted Augmentation of Nuclear Gene Output; LGMD: limb girdle muscular dystrophy; CMD: congenital muscular dystrophy; FCMD: Fukuyama congenital muscular dystrophy; CADASIL: cerebral autosomal dominant arteriopathy with subcortical infarcts and leukoencephalopathy; BPAN: beta-propeller protein-associated neurodegeneration.

### 4.3. Preclinical Research

#### 4.3.1. Neuromuscular Diseases

Splice-switching therapy is being investigated in many neuromuscular diseases (Table 2).

Fukuyama muscular dystrophy is a congenital muscular dystrophy caused by an abnormality in *FKTN* involved in the glycosylation of alpha-dystroglycan [66]. Most cases have a 3 kb retrotransposon (SVA) insertion in the 3′ untranslated region as the founder mutation. The SVA insertion creates a splice acceptor site, resulting in splicing defects, and therapeutic studies are underway to normalize splicing using AS-oligo [67]. Meanwhile, variants in intron 5, which are the second most common after the founder mutation, create splice donor sites and insert a pseudoexon into mRNA. In cultured myotube cells derived from patients, AS-oligo against ESE located within the pseudoexon inhibited pseudoexon insertion and improved alpha-dystroglycan glycosylation [26].

Basic research results of the induction of pseudoexon skipping by AS-oligo have also been reported in cases of beta-propeller protein-associated neurodegeneration (BPAN), in which a point mutation in intron 5 of the *WDR45* gene caused insertion of a pseudoexon [38], and in Ullrich congenital muscular dystrophy, in which a point mutation in intron 11 of the *COL6A1* gene caused insertion of a pseudoexon [25].

In addition, it was shown in cultured muscle cells of patients with limb-girdle dystrophy (LGMD) R2 with a truncating variant in exon 32 of the *DYSF* gene that AS-oligo can induce functional protein expression by inducing skipping of exon 32, which is in-frame [21].

The *SCN1* gene, which is responsible for Dravet’s syndrome, fails to produce functional proteins when exon 20N is inserted by alternative splicing [30]. A therapeutic strategy called Targeted Augmentation of Nuclear Gene Output (TANGO), which increases functional proteins by inhibiting exon 20N insertion via AS-oligo, is also being investigated for this disease caused by haploinsufficiency due to *SCN1A* variants [30].

Meanwhile, AS-oligo is also being investigated for triplet repeat diseases. In Huntington’s disease, which is caused by the elongation of CAG repeats in exon 1 of the *HTT* gene, the N-terminal fragment containing the elongated polyglutamine chain cleaved by caspase 6 is involved in disease progression [68]. Inducing the skipping of exon 12, the site of action of caspase-6, by AS-oligo has been reported as a possible therapeutic approach [29]. Meanwhile, fragile X syndrome is caused by elongation of the CGG repeat in the 5′ UTR of the *FMR1* gene, and the extension of this repeat induces the insertion of a pseudoexon in intron 1 [34]. The possibility of treatment by inhibiting the insertion of a pseudoexon using AS-oligo has been reported [34]. In myotonic dystrophy, CTG repeat elongation of the 3′ UTR of the *DMPK* gene induces splicing defects in other genes [69]. Exon 7a insertion into the *Clcn1* gene is known to be associated with myotonia, and it has been reported that inducing exon 7a skipping using AS-oligo against exon 7a splicing donor sites could be a therapeutic approach [27].

In addition to DMD and SMA, further development of splice-switching therapy in neuromuscular diseases is expected.

#### 4.3.2. Inborn Errors of Metabolism

Splice-switching therapy is also being investigated for inborn errors of metabolism (Table 2).

Glycogen storage disease type 1a is caused by a deficiency of glucose 6 phosphatase alpha (G6Pase-alpha encoded by *G6PC*) [70]. The c.648G>T variant, which is common in East Asia, activates a potential splicing acceptor site in exon 5 and induces splicing abnormalities. Studies in mouse models have shown that AS-oligo treatment against the potential splicing acceptor site can correct the splicing abnormality and ameliorate the pathology of this mutation [41].

In propionic acidemia, the c.1285-1416A>G variant in intron 14 of the *PCCA* gene inhibits ESS activity and promotes ESE activity, which results in pseudoexon insertion. AS-oligo can inhibit this pseudoexon insertion and thereby restore enzyme activity, as shown in an experimental system of cultured cells [45]. This pseudoexon is also found in the wild type, and AS-oligo may improve residual PCC activity in other mutation cases via its use as a TANGO [45].

In phenylketonuria caused by phenylalanine hydroxylase deficiency, a variant that induces exon 11 skipping has been reported to be inhibited by AS-oligo in a cultured cell system [44].

Further development of splice-switching therapy in inborn errors of metabolism is expected.

#### 4.3.3. Other Monogenic Diseases

In addition to neuromuscular diseases and inborn errors of metabolism, splice-switching therapy is being investigated for many other diseases.

In human bronchial epithelial cells with cystic fibrosis caused by a nonsense mutation within exon 23 of the *CFTR* gene, it was reported that induction of exon 23 skipping by AS-oligo restores the open reading frame, increases CFTR expression, and increases CFTR-mediated chloride current [64].

In renal diseases, it was reported that, in X-linked Alport syndrome caused by the nonsense variant c.1411C>T in exon 21 of the *COL4A5* gene, AS-oligo administration, which induces exon 21 skipping, produces a functional protein with an in-frame deletion in a mouse model [51]. In addition, phenotypic improvement as well as pathological improvement, such as the expression of collagen α5 chains in the glomerular and tubular basement membrane, was reported [51]. Moreover, in autosomal recessive polycystic kidney disease (ARPKD), activation of a potential splice acceptor site by c.2141-3T>C in intron 21 of the *PKHD1* gene was found to be regulated by AS-oligo in a minigene-based cultured cell system [52].

In skin diseases, AS-oligo was reported to restore protein expression in skin fibroblasts from patients with xeroderma pigmentosum, in which the c.207+196T>A mutation in intron 1 of the *ERCC4/XPF* gene results in the formation of a splice donor site and 192 bases in intron 1 are inserted between exons 1 and 2 [58]. In a case of recessive dystrophic epidermolysis bullosa with c.425A>G variant in the exon 3/intron 3 junction of the *COL7A1* gene, activation of a cryptic splicing donor site and exon skipping were induced. It was reported that AS-oligo transduction increased normal splicing products and restored type VII collagen expression in the fibroblasts in this case [60].

Meanwhile, in eye diseases, it was reported that transfection of AS-oligo and induction of exon 12 skipping in fibroblasts with retinitis pigmentosa 11 caused by nonsense variant within exon 12 of the *PRPF31* gene restored the open reading frame and increased PRPF31 expression [56]. Finally, in Stargardt disease, the most common hereditary form of maculopathy, basic studies of treatment using AS-oligo to correct splicing have also been reported [54,55].

The main studies on splice-switching treatment, including these diseases, are listed in Table 2.

## 5. The Expansion of Splice-Switching Therapy for Non-Monogenic Diseases

Splice switching therapy is being explored as a therapeutic strategy not only for monogenic diseases, but also for malignant tumors and immune-related diseases as shown below and summarized in Table 3. It has been reported that alternative splicing plays an important role in the formation and growth of tumors in many malignancies, and controlling such splicing is attracting attention as a therapeutic strategy [71].

In castration-resistant prostate cancer, it is known that the suppression of androgen receptor expression can control tumor progression. Meanwhile, androgen receptor produced from a splicing variant encompassing exon 3a, which uses a new splice donor site upstream of the original splice donor site of exon 3 of the *androgen receptor* (*AR*) gene, is inhibited from entering the nucleus [72]. It was reported that AS-oligo could promote exon 3a insertion, and cell proliferation was also inhibited in MTT assays [72].

Epidermal growth factor receptor is a protein involved in cancer cell proliferation, differentiation, and invasion, and regulation of splicing and suppression of function by AS-oligo could be a strategy for treating cancer [73]. It has been reported that AS-oligo can induce exon 3, exon 18, and exon 21 skipping in glioblastoma, liver cancer, and breast cancer cell lines, as well as suppress the migration potential of glioblastoma cells [73]. Meanwhile, the insulin receptor has a splice variant that skips exon 11 and is involved in the growth of rhabdomyosarcoma; it has been reported that AS-oligo can induce the inclusion of exon 11, thereby suppressing proliferation and migration [74].

Alternative exon 6 skipping of the *interleukin 7 receptor* (*IL7R*) gene results in the formation of soluble IL7R (sIL7R), and sIL7R expression is strongly associated with the development of autoimmune diseases such as multiple sclerosis (MS) [75]. It was shown that AS-oligo transfection of CD4^+^ T cells can promote exon 6 inclusion, thereby suppressing sIL7R expression. Meanwhile, another AS-oligo induced exon 6 skipping and promoted sIL7R expression, the former is a promising therapeutic strategy for MS and the latter for tumor immune-mediated [76].

IgE is intensely involved in the pathogenesis of allergic diseases, and the FcεRIβ (β-subunit of the high-affinity IgE receptor) encoded by the *MS4A2* gene has a short isoform that skips exon 3 [77]. In mouse bone marrow-derived mast cells, induction of exon 3 skipping by AS-oligo was reported to promote the production of the short isoform and suppress the expression of FcεRI on the cell surface, and AS-oligo administration to a mouse model of allergic dermatitis resulted in the improvement in symptoms [77].

The expression of cold-inducible RBM3, which is involved in brain protection, is promoted by the induction of skipping of exon 3a, which has a termination codon, at low temperatures, and the induction of exon 3a skipping with AS-oligo promoted RBM3 expression even at normal temperatures [78]. In addition, in a mouse model of prion disease, neuronal loss and cavernous hemangioma were prevented by the administration of AS-oligo. RBM3-inducing AS-oligo may be neuroprotective against diseases ranging from acute brain injury to Alzheimer’s disease [78].

In vascular smooth muscle, alternative splicing of *myosin phosphatase regulatory subunit 1* (*Mypt1*) causes exon 24 skipping, and Mypt1 produced by such skipping is involved in vasodilation. AS-oligo has been shown to induce exon 24 skipping in mice, and is a promising therapeutic strategy for cardiovascular disease [79].

Tissue fibrosis is a progressive pathology in many conditions, and AS-oligo is being investigated for the inhibition of fibrosis in renal, hepatic, and muscular tissues. Endoglin (ENG), a TGF-β co-receptor involved in the pathogenesis of interstitial fibrosis in chronic kidney disease, has two splicing variants: long-ENG, which undergoes normal splicing, and short-ENG, in which intron 14 is retained. The former promotes fibrosis, while the latter suppresses it [80]. Treatment of human renal fibroblast cell lines with AS-oligo induced intron 14 retention and decreased protein expression of αSMA, type I collagen, and fibronectin [81]. In addition, fibrosis in metabolic-dysfunction-associated steatohepatitis (MASH; previously termed NASH) involves alternative splicing of *TEAD1*, and exon 5 skipping promotes fibrosis. In mice, AS-oligo was found to induce the insertion of exon 5, which ameliorates liver fibrosis [82]. Periostin (*Postn*), which is an enhancer of TGF-β1-induced fibrosis, is a protein involved in the fibrosis of skeletal muscle and other tissues, and its exon 17-skipping splicing variant suppresses fibrosis. Moreover, in a mouse model of DMD, administration of AS-oligo, which induces exon 17 skipping, reduced collagen accumulation [83].

Thus, splicing-switch therapy is expanding not only in monogenic diseases but also in non-monogenic ones (Table 3).

**Table 3:** Splice switching therapy with antisense nucleic acids for non-monogenic disease.

Disease	Gene	Exon	Splice Switching	Phase	Author, Year
glioblastoma	*EGFR*	3, 18, 21	ES	in vitro	Balachandran et al., 2023 [73]
prostate cancer	*AR*	I3	IR	in vitro	Wang et al., 2023 [72]
	*ERG*	4	ES	mouse	Li et al., 2020 [84]
rhabdomyosarcoma	*INSR*	11	EI	mouse	Khurshid et al., 2022 [74]
breast cancer	*BRCA1*	11	ES	in vitro	Smith et al., 2017 [85]
oral cancer	*PD-1*	3	ES	in vitro	Wang et al., 2024 [86]
cancer	*CD44*	v8	ES	in vitro	Fukushima et al., 2020 [87]
multiple sclerosis/cancer	*IL7R*	6	EI/ES	in vitro	Galarza-Muñoz et al., 2022 [76]
multiple sclerosis	*ITGA4*	4	ES	mouse	Aung-Htut et al., 2019 [88]
allergy	*MS4A2*	3	ES	mouse	Cruse et al., 2016 [77]
acute brain injury and Alzheimer’s disease	*RBM3*	3a	ES	mouse	Preußner et al., 2023 [78]
diabetes mellitus	*MSTN*	2	ES	mouse	Eilers et al., 2021 [89]
muscle fibrosis	*Postn*	17	ES	mouse	Trundle et al., 2024 [83]
	*ALK5*	2	ES	mouse	Kemaladewi et al., 2014 [90]
liver fibrosis	*TEAD1*	5	EI	mouse	Isaac et al., 2024 [82]
renal fibrosis	*ENG*	int 14	IR	in vitro	Gerrits et al., 2024 [81]
vascular dysfunction	*Mypt1*	24	ES	mouse	Damacena de Angelis et al., 2024 [79]
Dupuytren’s disease	*ALK5*	2	ES	in vitro	Karkampouna et al., 2014 [91]
hypertrophic scar	*ALK5*	2	ES	in vitro	Raktoe et al., 2020 [92]

IR: retention of intron. Other abbreviations are the same as in Table 2.

## 6. Conclusions and Perspectives

It has been 30 years since we proposed splice-switching therapy for DMD, in which splicing is modified by AS-oligo to express functional proteins. Various strategies are being considered for splice-switching therapy, including exon inclusion, pseduexon inhibition, and correction of aberrant splicing by mutation, rather than exon skipping alone. Even in monogenic diseases, modifying the pathology caused by the mutation is also being considered, as in myotonic dystrophy, Pompe disease, and familial hypercholesterolemia, rather than only modifying the mutation responsible for the disease [27,42,50].

In addition, modified nucleic acids are used to enhance the efficacy of AS-oligos. Nusinersen, a drug for SMA used in clinical practice, is a 2′-*O*-methoxyethyl (MOE) modified AS-oligo, and eteplirsen, golodirsen, viltolarsen, and casimersen, drugs for DMD, are phosphorodiamidate morpholino (PMO) AS-oligos [93]. In the studies shown in Table 2 and Table 3, AS-oligos with 2′-*O*-methyl modification [21,22,24,26,31,35,36,44,45,48,50,53,57,62,73,90], MOE modification [18,25,27,34,40,54,60,64,65,72,78,81,86], PMO [33,39,43,49,56,58,76,83,84,85,89], and 2′-*O*, 4′-*C*-ethylene-bridged nucleic acid (ENA) [38,51,87], among others, were also used, and it is expected that modified nucleic acids with even higher efficacy will be developed in the future.

Many splice-switching therapies are specific to the particular genetic variant, some of which are being developed for single cases and are attracting attention as N-of-1 treatment [18,19]. TANGO, in contrast, ameliorates loss of function by shifting the inherently accepted selective splicing in a more functional direction, a strategy that can target many mutations [30,45]. Splice-switching therapy is expected to continue to be deployed in a variety of ways.

Initially conceived as a personalized medicine for monogenic diseases, it is now being considered as a therapeutic strategy not only for monogenic diseases but also for non-monogenic ones such as cancer, immune-related diseases, and fibrosis.

Although adverse events are not currently a major problem in clinical practice, continued investigation of new adverse events, such as off-target effects, in long-term or high-dose use is warranted. It is hoped that the findings of these basic investigations of such therapies will be applied for new treatments in a clinical context, while further investigations into safety also need to be carried out.

## Figures and Tables

**Table 1:** Therapeutic strategies using splice-switching with antisense oligonucleotide [4].

1. Splice switching for responsible genes
(a) Induction of exon skipping
Correction of amino acid reading frame by inducing out-of-frame exon skipping
Induction of skipping of in-frame exons where nonsense mutations occur
Induction of pseudoexon skipping
Conversion of protein function by inducing exon skipping
(b) Induction of exon inclusion
(c) Inhibition of splice sites generated by mutations
2. Induction of splicing switches in genes other than the responsible gene

## Data Availability

No new data were created or analyzed in this study.
